# Water and nitrogen availability co-control ecosystem CO_2_ exchange in a semiarid temperate steppe

**DOI:** 10.1038/srep15549

**Published:** 2015-10-23

**Authors:** Xiaolin Zhang, Yulian Tan, Ang Li, Tingting Ren, Shiping Chen, Lixin Wang, Jianhui Huang

**Affiliations:** 1 State Key Laboratory of Vegetation and Environmental Change, Institute of Botany, the Chinese Academy of Sciences, Beijing 100093, China; 2 University of Chinese Academy of Sciences, Beijing 100049, China; 3 Department of Earth Sciences, Indiana University-Purdue University Indianapolis (IUPUI), Indianapolis, Indiana 46202, USA

## Abstract

Both water and nitrogen (N) availability have significant effects on ecosystem CO_2_ exchange (ECE), which includes net ecosystem productivity (NEP), ecosystem respiration (ER) and gross ecosystem photosynthesis (GEP). How water and N availability influence ECE in arid and semiarid grasslands is still uncertain. A manipulative experiment with additions of rainfall, snow and N was conducted to test their effects on ECE in a semiarid temperate steppe of northern China for three consecutive years with contrasting natural precipitation. ECE increased with annual precipitation but approached peak values at different precipitation amount. Water addition, especially summer water addition, had significantly positive effects on ECE in years when the natural precipitation was normal or below normal, but showed trivial effect on GEP when the natural precipitation was above normal as effects on ER and NEP offset one another. Nitrogen addition exerted non-significant or negative effects on ECE when precipitation was low but switched to a positive effect when precipitation was high, indicating N effect triggered by water availability. Our results indicate that both water and N availability control ECE and the effects of future precipitation changes and increasing N deposition will depend on how they can change collaboratively in this semiarid steppe ecosystem.

The East Asian summer rainfall has already extended its distribution northward to the northern China, and its increasing amount will range from 93 to 136 mm by the end of this century^1^. This will influence ecosystem CO_2_ exchange (ECE) by affecting both C assimilation through plant photosynthesis and C release by plant and soil respiration especially in those arid and semiarid areas. Owing to the inconsistent changes in both magnitude and direction of gross ecosystem photosynthesis (GEP) and ecosystem respiration (ER), some previous studies have shown positive changes in net ecosystem productivity (NEP) to increasing precipitation^2^,^3^,^4^, but others show no changes^5^,^6^ or even negative changes^7^ in different ecosystems. There are a great number of studies investigated the effects of water addition on NEP, but most of them only focused on short-term effects. Thus, experiments with relatively longer time responses of ECE to water addition are needed, especially in areas with remarkably interannual variations in precipitation^8^.

Carbon fluxes in the future will face the precipitation changes including not only those in summer rainfall amount and rainfall pattern, but also those in snow amount, and melting time. Snow plays an important role controlling ECE in winter and the following growing seasons^9^. Snowfall is an important precipitation form in northern China and will increase when cold wave events occur owing to the increasing water vapor amount from the north^10^. Generally, snow cover can last five months in Inner Mongolia grasslands and protect soil from harsh conditions by weakening fluctuation of soil temperature during winter. Snowmelt promotes soil moisture that is in favor of plant growth^11^ and may have critical effects on plant growth during the entire growing season especially when plants begins to germinate^9^,^12^. However, melting of snow can also take away nutrients from soil, which often happens during plants’ initial growth under the continental climate^13^,^14^. Therefore, how future snowfall affects ECE in the semiarid temperate steppe remains unclear.

Global N deposition is expected to increase under the background of the climate change caused by intensive anthropogenic activities^15^. Nitrogen deposition is likely to increase soil N availability which will stimulate plant growth as studies show that N is a main constraint for plant metabolism in many water limited areas^16^,^17^. However, there is no consensus on effects of N deposition on ECE, as some studies suggest that it can stimulate ecosystem CO_2_ assimilation^8^,^18^, while others show no effects or even negative effects^19^,^20^,^21^. There is no consensus on the NEP responses to N deposition either, and clearly more studies are needed, especially in the semiarid steppe ecosystems.

As both water and N are important limiting factors in the semiarid temperate steppe, changes in precipitation and N deposition at the same time may potentially induce complex interactions on ecosystem structure and functioning. Burke *et al.*^22^ has demonstrated that water addition can promote net N mineralization and consequently increase soil N availability. Therefore water addition indirectly affects ECE through its impact on N availability^23^. However, N availability is also sensitive to precipitation through nitrate leaching and gas losses through denitrification^24^. Thus, it is difficult to predict whether increase in both water and N promote each other to generate greater effects on NEP especially in the semiarid areas.

The semiarid temperate steppe in northern China is an important component of the Eurasian steppe. The steppe is not only sensitive to climate change^25^ but also limited by water and N^16^. To address the uncertainties of interactive water and N effects on ECE, we conducted a manipulative experiment including water addition (either through spring snow or through summer rainfall) and N addition in a semiarid temperate steppe to examine how ECE will respond to changing precipitation and increasing N deposition. We hypothesized that (1) spring snow addition would increase ECE due to its effect of maintaining soil temperature in winter and relieving water limitation at the beginning of growing season; (2) additional summer water would increase ECE since it increases water supply for plant growth; (3) N addition could enhance ECE because of N limitation on plant growth in this study area. Based on these hypotheses, interactions between spring snow or summer water and N addition on ECE are expected since N will become more available with increase of water supply.

## Results

### Interannual variation in precipitation and soil microclimate

**Figure 1:**
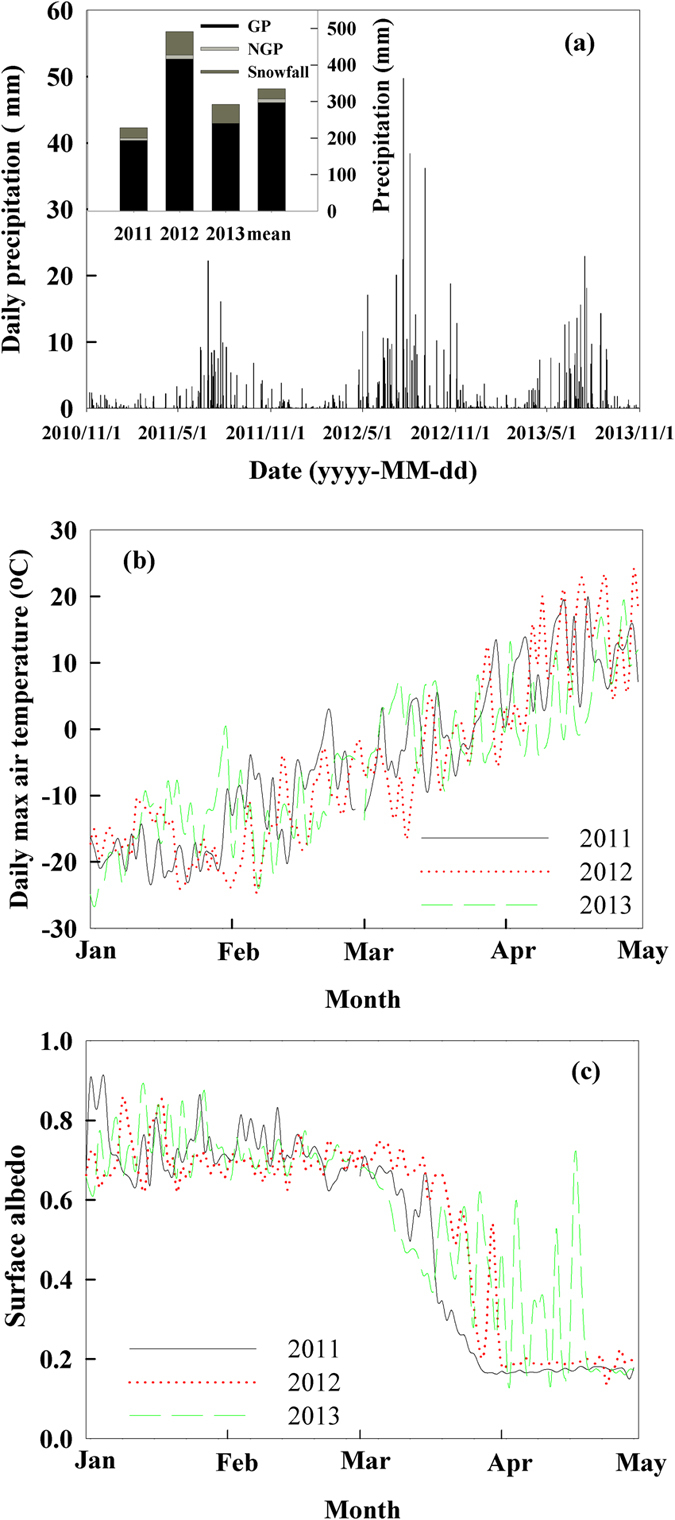
Daily snowfall or rainfall (a) from November 2010 to October 2013 with inset figure of snowfall (from previous year Nov.), rainfall in the growing season (GP, from May to Oct.), rainfall in the non-growing season (NGP, Apr.) in 2011, 2012, 2013 and mean from 1982 to 2013, respectively. Daily maximum air temperature (**b**) and surface albedo in the daytime (**c**) are also shown from January to April in 2011, 2012 and 2013. Data of rainfall and surface albedo is from an adjacent eddy flux tower, and snowfall amount is described as water equivalent of snow.

Precipitation showed remarkably different patterns and amount across the three study years (Fig. 1a). The annual natural precipitation amount (from previous November to October) was 231.7 mm in 2011, 464.7 mm in 2012, and 320.3 mm in 2013, respectively (Fig. 1a). The rainfall amount during the growing season (May through October) was 193.4 mm in 2011, 416.3 mm in 2012, and 242.7 mm in 2013 (Fig. 1a inset). The snowfall (from previous November to April) was 31.9 mm in the 2010–2011 snowing period. The snowpack melted away around March 16 based on the data of daily maximum air temperature and surface albedo (Fig. 1b,c). Similarly, the snowfall was 37.9 mm in the 2011–2012 snowing period and the snow melted away around April 1 (Fig. 1a–c). The snowfall was 77.6 mm in the 2012–2013 snowing period. Among them, 25.6 mm happened in April and the snow melted away around April 16 (Fig. 1a–c), resulting in snow cover being almost one month longer than that in 2011.

**Figure 2:**
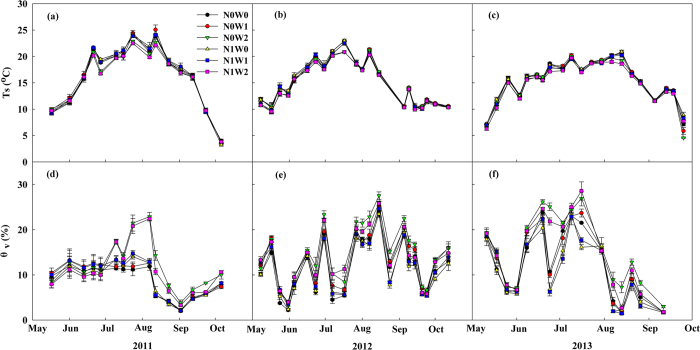
Seasonal variations of soil temperature (Ts) (a–c) and soil moisture (θv, V/V%) (d–f) in all the treatments of 2011, 2012 and 2013. Values represent mean ± 1SE (n = 5).

Across the three growing seasons, water addition had generally negative effects on soil temperature but positive effect on soil moisture. Spring snow addition decreased soil temperature by 0.2 °C in 2012 and 0.1 °C in 2013, but showed no significant effects in 2011 (Fig. 2a–c, Table 1). Spring snow addition had increased soil moisture by 6.4% in 2011 (*P* = 0.04), 9.2% in 2012 (*P* < 0.001), while the increase was only marginally significant in 2013 (by 3.0%, *P* = 0.10) (Fig. 2d–f, Table 1). Summer water addition significantly elevated soil moisture by 31.5% in 2011, 22.2% in 2012 and 31.0% in 2013, but lowered soil temperature by 0.5 °C in 2011, 0.4 °C in 2012 and 0.3 °C in 2013 (Fig. 2a–f).

Nitrogen addition had either non-significant or even negative effects on soil temperature and moisture regardless of water addition (i.e., spring snow addition or summer water addition). Nitrogen addition had significantly negative effect on soil temperature in 2013 (by decreasing 0.2 °C, *P* < 0.001) in the spring snow addition treatment while no significant effect in the other two years. Nitrogen addition decreased soil temperature significantly in the summer water addition treatment by 0.2 °C in 2012 (*P* = 0.004), 0.3 °C in 2013 (*P* < 0.001) while had insignificant effect in 2011 (*P* = 0.30, Table 1). Nitrogen addition also decreased soil moisture in 2012 and 2013 in both spring snow addition (by 7.0% in 2012, *P* < 0.001, and 11.4% in 2013, *P* < 0.001) and summer water addition treatment (by 5.2% in 2012, *P* < 0.001, and 8.5% in 2013, *P* < 0.001), but showed no significant effect in 2011 regardless of spring snow addition (*P* = 0.33) or summer water addition (*P* = 0.15) (Fig. 2a–f, Table 1). Besides, N addition had significant interactive effects with spring snow addition on soil moisture in 2012 (by decreasing 1.4%, *P* = 0.007) and marginally significant effect on soil temperature in 2013 (by decreasing 0.3 °C, *P* = 0.07). Nitrogen addition also had significant interactive effects with summer water addition on soil temperature in 2012 (by decreasing 0.6 °C, *P* = 0.007) and 2013 (by decreasing 0.7 °C, *P* < 0.001) and on soil moisture in 2011 (by increasing 27.5%, *P* = 0.02) (Fig. 2b–d, Table 1).

### Effects of water and N addition on ecosystem CO_2_ exchange

**Figure 3:**
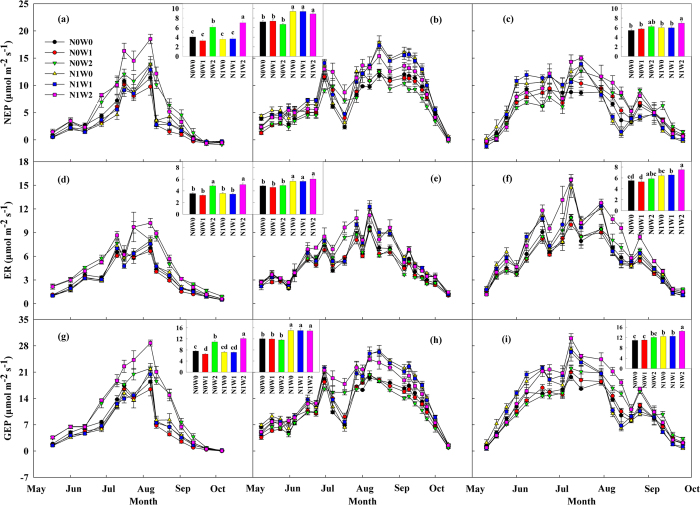
Seasonal dynamics of net ecosystem productivity (NEP) (a–c), ecosystem respiration (ER) (d–f) and gross ecosystem photosynthesis (GEP) (g–i) in 2011 (left panels), 2012 (middle panels), and 2013 (right panels) respectively with averages being given in the inset figures. Values represent mean ± 1SE (n = 5). Different letters in the inset figures indicate significant differences (*P* < 0.05) among six treatments.

Spring snow addition only significantly affected ECE in the year that precipitation was below the normal. In 2011, spring snow addition significantly decreased ER (6.0%, *P* = 0.004) and GEP (7.3%, *P* = 0.009) while only marginally decreased NEP (9.0%, *P* = 0.08) (Fig. 3a,d,g, Table 1). There was only marginally significant effect for ER (by decreasing 2.7%, *P* = 0.07) in 2012 while there was no significant effect for the all three fluxes in 2013 (Table 1).

Summer water addition significantly increased ECE (NEP, ER and GEP) in years when the natural precipitation was normal (2013) or below normal (2011), but the effect was only significant for NEP and ER when the natural precipitation was above normal (2012) (Fig. 3, Table 1). Summer water addition enhanced NEP, ER, and GEP in 2011 (by 73.6%, 39.6% and 56.1%, respectively, *P* < 0.001 for all cases) (Fig. 3a,d,g), and in 2013 (by 15.2%, 11.7%, and 13.4%, respectively, *P* < 0.001 for all cases) (Fig. 3c,f,i). However, it decreased NEP (by 6.4%, *P* < 0.001) while increased ER (by 4.1%, *P* = 0.005) and had insignificant effect on GEP in 2012 (Fig. 3b,e,h).

Nitrogen addition effects on ECE (NEP, ER, and GEP) under both spring snow addition and summer water addition treatments varied with natural precipitation in the three years. Nitrogen addition enhanced NEP, ER and GEP in association with spring snow addition by 29.0%, 19.3% and 25.1% (*P* < 0.001 for all cases) respectively in 2012 (Fig. 3b,e,h) and by 7.4%, 20.0% and 13.5% in 2013 (*P* = 0.03, *P* < 0.001, and *P* < 0.001, respectively) (Fig. 3c,f,i) while the effect was not significant in 2011 (Table 1). Nitrogen addition enhanced NEP, ER and GEP in association with summer water addition by 31.7%, 19.3% and 26.6% (*P* < 0.001 for all cases) respectively in 2012 (Fig. 3b,e,h) and by 11.2%, 22.4% and 16.7% in 2013 (*P* < 0.001 for all cases) (Fig. 3c,f,i) while the effect was not significant in 2011 (Table 1).

The interactive effect between N addition and spring snow addition was only found significant in 2011 on NEP (by increasing 10.6%, *P* = 0.05) and GEP (by increasing 5.9%, *P* = 0.02), and in 2012 on ER (by decreasing 16.0%, *P* = 0.08), while insignificant for all three variables in 2013 (Table 1). The interactive effect between N addition and summer water addition was found significant in 2011 for NEP (by increasing 73.5%, *P* = 0.005) and GEP (by increasing 59.5%, *P* = 0.004), in 2012 for ER (by increasing 24.1%, *P* = 0.04), and in 2013 for ER (by increasing 36.3%, *P* = 0.002) while only marginally significant in 2013 for GEP (by increasing 32.3%, *P* = 0.06) (Table 1).

**Figure 4:**
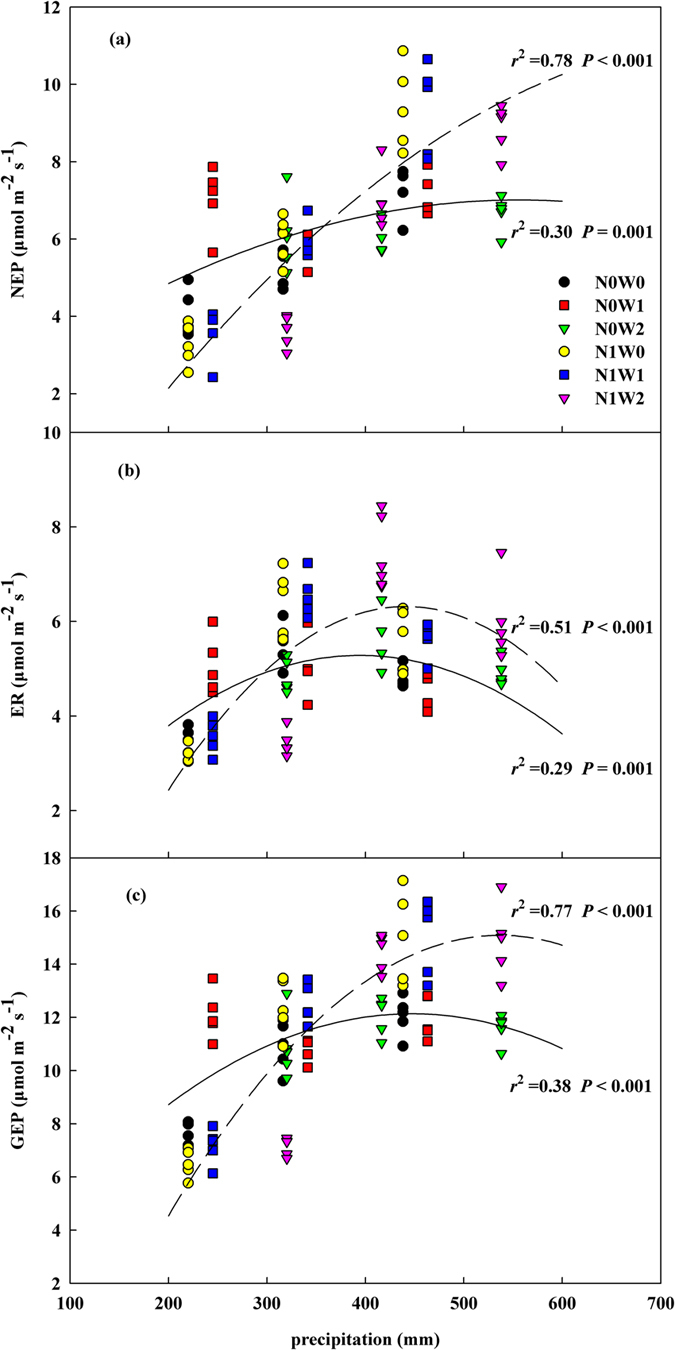
Dependence of net ecosystem productivity (NEP, (a)), ecosystem respiration (ER, (b)), and gross ecosystem photosynthesis (GEP, (c)) on precipitation (natural rainfall + water addition) in plots without N addition (solid line) and with N addition (dashed line) in control (N0W0, black circle), spring snow addition (N0W1, red square), summer water addition (N0W2, green triangle), nitrogen addition (N1W0, yellow circle), spring snow with nitrogen addition (N1W1, blue square), summer water with nitrogen addition (N1W2, pink triangle) treatments. The empirical equations for NEP, ER and GEP in plots without N addition were y = (−1.71E−05)*x^2^ + 0.019*x + 1.733, y = (−3.93E−05)*x^2^ + 0.031*x−0.83 and y = (−5.59E−05)*x^2^ + 0.05*x + 0.956, respectively and in plots with N addition were y = (−2.59E−05)*x^2^ + 0.041*x−5.021, y = (−6.69E−05)*x^2^ + 0.059*x−6.692 and y = (−9.32E−05)*x^2^ + 0.1*x−11.736, respectively.

ECE, including NEP, ER and GEP, had a quadratic relationship with total precipitation (manipulative + natural) in plots pooled either with or without N addition. The NEP, ER, GEP reached their highest values at the total precipitation of 554.3 mm (*r*^2^ = 0.30, *P* = 0.001), 394.3 mm (*r*^2^ = 0.29, *P* = 0.001) and 447.3 mm (*r*^2^ = 0.38, *P* < 0.001), respectively, in plots without N addition while at the total precipitation of 793.0 mm (*r*^2^ = 0.78, *P* < 0.001), 441.3 mm (*r*^2^ = 0.51, *P* < 0.001) and 536.6 mm (*r*^2^ = 0.77, *P* < 0.001) in plots with N addition (Fig. 4a–c). When the precipitation was low, NEP, ER and GEP were higher in plots without N addition than with N addition, but the trend became opposite when the precipitation was high (Fig. 4a–c). We calculated the thresholds in the precipitation amount, and the results were 357.6 mm, 294.9 mm, and 340.1 mm for NEP, ER, and GEP, respectively (Fig. 4a–c).

## Discussion

### Precipitation increase causes limited change of ecosystem CO_2_ exchange

Precipitation change may appear in both snowfall and rainfall especially in those middle to high latitude areas, which may result in different effects on ECE^26^. However, results from previous studies varied remarkably among various ecosystems studied and ecological processes measured.

Snow addition not only leads to warmer, wetter, relatively C substrate-rich soils under snowpack^27^, but also increases soil water availability and decreases soil temperature after snowmelt as shown in this study (Table 1). Decrease in soil temperature after snowmelt could reduce ECE while increase in soil water content could enhance ECE (Fig. S2). Thus, the net effects of increased snowfall on ECE mainly depend on how large the contrasting effects are. Significant positive effects of winter snow addition on ECE (NEP, GEP and ER) were reported in a mixed-grass prairie in the United States^28^. However, both positive and negative effects of spring snow addition were found in this study on the three variables, and they also varied remarkably among years. Besides, the effect of spring snow addition was not strong enough to impact the aboveground net primary productivity (ANPP) and belowground biomass (BGB) across the three years (Table S1). Our findings indicate that the increase in spring snow can play an important role but may have limited effects on the ECE in the temperate steppe ecosystems because snowfall in this area only accounts for a small portion (12% in average) of the total annual precipitation.

Our results also showed that ECE were all significantly enhanced by summer water addition across the three growing seasons, but the effect was related to natural precipitation amount. The positive effect of summer water addition on ECE was mostly common in previous studies in this semiarid region^2^,^8^ and in other temperate grasslands^29^, the non-significant and even negative effect induced by increased rainfall was rarely reported before. Our results indicate that precipitation increase, especially rainfall, will enhance ECE but may finally hinder ECE especially facing extreme events in the future by the way of changing plant growth. We also found an increase in ANPP as in 2013, a decrease as in 2012, and no significant change as in 2011 (Table S1). However, summer water addition all significantly increased BGB in the three years. Our results indicate that ECE changes may be decoupled from ANPP changes, while BGB changes are consistent with ECE in this semi-arid steppe ecosystem.

### Water and N availability co-control ECE

Soil N availability may be significantly controlled by soil water availability^30^,^31^. Low water availability constrains soil N mineralization and effectiveness of N in soil^16^,^17^,^29^,^32^,^33^ as soil moisture primarily adjusts the availability of inorganic N via water dependence of microbial activities, and their uptake by plants. Our results showed that N addition had remarkably different effects on ECE across the three growing seasons, and was highly dependent not only on the experimental precipitation addition but also on the natural precipitation amount. There was negative (though insignificant) response in ECE to N addition in 2011 when precipitation was below normal (Fig. 4). Previous studies in this area^34^ and in African savanna^35^,^36^ showed that N addition did not significantly affect ECE when precipitation was low. Nitrogen addition showed significant increasing effects on all three ECEs in both 2012 and 2013 when the annual precipitation was well above (in 2012) or around normal (in 2013) regardless of water forms, which was largely in agreement with results from previous studies^8^,^18^. Nitrogen addition increased ANPP by 39.6% in 2013 and marginally increased ANPP by 14.2% in 2011 and by 26.0% in 2012 but had no significant effect on BGB across the three years (Table S1). Our results suggest that positive effects of N deposition on ECE and ANPP are modulated more by natural precipitation likely through affecting plant growth. However, changes of BGB are primarily controlled by water availability instead of N availability.

However, the difference in the responding pattern of NEP, ER and GEP to precipitation change between N addition and without N addition suggests that the sensitivity of ECE to N addition has increased with increasing precipitation (Fig. 4a–c). The switching N addition effect on ECE from negative to positive indicate that increasing N deposition may inhibit ECE when precipitation is lower than normal but will enhance ECE when precipitation is above normal. The reason causing inhibition of ECE is in fact not clear. Ammonia toxicity was once believed to reduce plant growth^37^ and to impede microbial activities to reduce soil respiration^38^,^39^. However, in this study, nitrates rather than ammonia were significantly higher in N fertilization plots indicating nitrification was generally strong or leaching is potentially low in 2011 when precipitation was low (Table S2). Besides, the turning points occur around the long-term mean annual precipitation but were slightly different among the three variables with NEP requiring higher precipitation, ER lower precipitation, and GEP being in between, which may indicate that both plant system and soil microbial system in this semiarid steppe have adapted to the long-term average climate.

### N addition raised the responding threshold to precipitation

Previous studies in the study area showed that ECE increased either linearly^8^ or quadratically^18^ with precipitation. Our results displayed that there was saturation in the response of ECE to precipitation, and extra precipitation may in fact have an inhibitive effect on ECE (Fig. 4a–c). Besides, N addition could raise remarkably the responding threshold of ECE to precipitation especially for NEP flux, but it did not alter the optimum responding pattern (Fig. 4).

Moreover, our results showed that NEP, ER, and GEP approached their peak values at different precipitation amount (Fig. 4a–c), which was clearly different from the results of those previous studies^8^,^18^ regardless of responding patterns. Our results indicate that the three ecosystem processes (NEP, ER, and GEP) are controlled by different factors, which are influenced by water availability to different extents. NEP is primarily determined by N uptake by plants in the semiarid typical steppe^40^, while ER largely by microbial respiration^41^. Plant N uptake and microbial activities may respond to soil water availability differentially at both speed and magnitude^41^ resulting in the three ECE processes changing asynchronously with precipitation (Fig. 4).

The remarkably higher response threshold to precipitation for NEP than for ER, especially with N addition, indicates that both N deposition and precipitation increase are prerequisites for enhancing C sequestration in such a semiarid temperate steppe. Thus, the combined effects of N deposition and possible precipitation change under future global change scenarios in northern China will become critical factors that should be considered in ecological modeling to project the future states of C cycling in this area^42^.

## Conclusions

Through a factorial water (either in rainfall or snow form) and N addition experiment in a semiarid typical steppe in the northern China, we found that water addition, especially summer water addition, showed complex effects on ECE depending on ECE components when the natural precipitation was above normal. It had significantly positive effects on ECE (NEP, ER, and GEP) in years when the natural precipitation was normal or below normal, but the effect was trivial and even negative for GEP when the natural precipitation was above normal because high precipitation had a significantly increasing effect on ER but a decreasing effect on NEP. On the contrary, N addition showed strong positive effects on ECE in years when the natural precipitation was normal or above normal while the effect was minimum when the natural precipitation was below normal. Ecosystem CO_2_ exchange can increase significantly with precipitation, but will be inhibited when precipitation exceeds a certain threshold. Besides, the threshold values in precipitation amount can be remarkably raised by N addition, especially for NEP. Our results implicate that the effect of increasing N deposition on C sequestration may highly depend on water availability while the effect of increasing precipitation on C sequestration requires higher N availability in the semiarid or arid steppe in northern China.

## Material and Methods

### Site description

The experiment site, located in the Inner Mongolia Grassland Ecosystem Research Station, Institute of Botany, the Chinese Academy of Sciences (43°33′N, 116°40′E, 1251 m a. s. l), is covered with a typical steppe. The dominant species include *Stipa grandis* and *Leymus chinensis*. The soil at the site belongs to Chernozem according to the Chinese soil taxonomic system, or is classified as Ustoll based on the US Soil Taxonomy^43^. The mean annual temperature (MAT) is 0.4 °C and the mean annual precipitation (MAP) is 333.3 mm (1982–2013), with 8.1% occurring in the form of snow in the snowing period generally spanning from November to next March, and 88.5% in the form of rainfall happening during the growing season from May to October, while the remaining 3.4% appeared in April. We considered a normal year when precipitation was close to the long term mean value while below normal or above normal when precipitation was lower or higher than the mean value.

### Experimental design and treatments

The experiment used a randomized block design with two levels of N addition (control, and N addition) interacted with two levels of water addition (control, and either spring snow addition or summer water addition) with 5 replicates per treatment resulting in thirty plots in total, each with an area of 25 m^2^ (5 m × 5 m) and at least 1 m walkway between two adjacent plots. Treatments were labeled as following: control (N0W0), spring snow addition (N0W1), summer water addition (N0W2), N addition (N1W0), spring snow with N addition (N1W1), summer water with N addition (N1W2) (see Fig. S1 in the Supporting Information). Nitrogen addition treatment was conducted at the early July every year since 2009, and 10 g N m^−2^ in the form of urea was applied in each N addition plot. The snowfall from 1982 to 2009 ranged from 11.1 to 64.0 mm with its mean value as 25.2 mm. As snowfall will increase in the northern China and an amount of 25 mm water equivalent of snow was added to each spring snow addition plot in early March every year since 2010. Spring snow was moved from the nearby area and then added evenly into the treatment plots. IPCC SRES B2 Scenarios also predicted that summer rainfall in the northern China would increase about 30% in the future (2071–2100)^1^. Total summer water addition amount in this study was 100 mm year^−1^ to each summer water addition plot and it was conducted from June 15 every year since 2010, and 10 mm water was applied weekly. With the spring snow addition, summer water addition and natural precipitation across the three study years, we designed an annual precipitation gradient with which changes in ecosystem C exchange (ECE) could be examined.

### Ecosystem CO_2_ exchange

Measurements of ECE, including net ecosystem productivity (NEP), ecosystem respiration (ER) and gross ecosystem photosynthesis (GEP), were performed between 8:30 and 10:30 am on the third or fourth sunny day after the summer water addition. We made three measurements every month in 2011 and once every week in 2012 and 2013 during the growing seasons from May to October. ECE were determined by an infrared gas analyzer (IRGA; LI-840, LI-COR Inc., Lincoln, NE, USA) with a transparent chamber (0.5 × 0.5 × 0.5 m) which both attached to an air pump (LI-COR Inc.). When conducting measurements the chamber was placed on a square stainless steel frame (0.5 × 0.5 m) which was inserted 5 cm into the soil at the center of each plot in 2010. In addition, the chamber had two small fans in upper opposite corners to mix air and a temperature probe to determine the air temperature inside chamber during measurements. Each measurement lasted about 80 seconds at 1 Hz frequency to attain CO_2_ concentration data for NEP calculation. When the inside of the chamber was vented completely after NEP measurements, a shade cloth covered the chamber to determine the respiration using similar measurements as ER. NEP and ER were calculated according to Equation 1 provided by Chen *et al.*^2^ and Jasoni *et al.*^44^


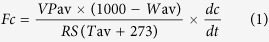


where *Fc* is the CO_2_ flux representing NEP and ER (μmol m^−2^ s^−1^); *V* is the volume of chamber (m^3^); *P*av, *W*av and *T*av are the average pressure (kPa), water mole fraction (mmol mol^−1^) and average temperature (°C) during the measurement period; *R* is the ideal gas constant (8.314 J mol^−1^K^−1^); *S* is the surface area covered by chamber (m^2^); and dc/dt is the slope of least squares linear regression of CO_2_ concentration on time. The sum of NEP and ER was used to calculate GEP as Equation 2.


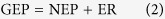


### Soil temperature and soil moisture

Soil temperature was determined at the depth of 10 cm with a thermocouple probe (LI 8100-201), which was continuously monitored at a 1 minute interval to calculate the means. At the same time, soil moisture was measured at the depth of 10 cm with a TDR-200 probe (Spectrum Technologies Inc., Plainfield, IL, USA) and was determined by the average three measurements.

### Statistical analysis

Mixed model analysis was applied to examine the major and interactive effects of either spring snow addition or summer water addition, and N addition during the growing seasons on soil temperature, soil moisture content, NEP, ER, and GEP. Linear or nonlinear regression analysis was employed to explore the relationships of ECE with soil temperature in the growing seasons, soil moisture in the growing seasons, and annual precipitation across the three years. All statistical analyses were performed using SPSS 16.0 for windows (SPSS Inc., Chicago, IL, USA).

## Additional Information

**How to cite this article**: Zhang, X. *et al.* Water and nitrogen availability co-control ecosystem CO_2_ exchange in a semiarid temperate steppe. *Sci. Rep.* **5**, 15549; doi: 10.1038/srep15549 (2015).

## Supplementary Material

Supplementary Information

## Figures and Tables

**Table 1:** 

		Spring snow addition treatment					Summer water addition treatment				
		Ts	*θv*	NEP	ER	GEP	Ts	*θv*	NEP	ER	GEP
2011	W	0.17	0.04	0.08	0.004	0.009	0.04	<0.001	<0.001	<0.001	<0.001
	N	0.82	0.33	0.78	0.11	0.82	0.30	0.15	0.36	0.34	0.22
	W*N	0.21	0.91	0.05	0.26	0.02	0.94	0.02	0.005	0.52	0.004
2012	W	0.003	<0.001	0.64	0.07	0.75	<0.001	<0.001	<0.001	0.005	0.11
	N	0.88	<0.001	<0.001	<0.001	<0.001	0.004	<0.001	<0.001	<0.001	<0.001
	W*N	0.72	0.007	0.58	0.08	0.84	0.007	0.15	0.99	0.04	0.40
2013	W	0.03	0.10	0.42	0.66	0.66	<0.001	<0.001	<0.001	<0.001	<0.001
	N	<0.001	<0.001	0.03	<0.001	<0.001	<0.001	<0.001	0.001	<0.001	<0.001
	W*N	0.07	0.56	0.35	0.11	0.98	<0.001	0.51	0.71	0.002	0.06

Results (*P*-values) of mixed model analysis on effects of water addition (W) as either spring snow addition or summer water addition, nitrogen addition (N), and their interactions on soil temperature (Ts, °C), soil moisture (*θv*, V/V%), net ecosystem productivity (NEP), ecosystem respiration (ER), and gross ecosystem photosynthesis (GEP) in 2011, 2012 and 2013.
