# Supplementary material for: Water and nitrogen availability co-control ecosystem CO2 exchange in a semiarid temperate steppe
Source: Sci Rep. 2015 Oct 23;5:15549. doi: 10.1038/srep15549 (PMC4616041; doi:10.1038/srep15549)
Supplement: Supplementary Information [file srep15549-s1.pdf]

Water and nitrogen availability co-control ecosystem CO_2 exchange in a semiarid temperate steppe

Xiaolin Zhang[1,2], Yulian Tan[1], Ang Li[1], Tingting Ren[1], Shiping Chen[1], Lixin Wang[3],

Jianhui Huang[1,*]

Supporting information

Fig. S1 The layout of the experiment site. Treatment codes: N0W0, control (CK); N0W1, addition of spring snow; N0W2, addition of summer water; N1W0, addition of nitrogen (N); N1W1, combined addition of N and spring snow; N1W2, combined addition of N and summer water.

Fig. S2 Relationships of net ecosystem CO_2 productivity (NEP), ecosystem respiration (ER) and gross ecosystem photosynthesis (GEP) with dynamics of soil temperature (Ts) (a, b, c) and soil moisture (θv, V/V%) (d, e, f) in all the treatments across the three growing seasons. The letter ns means not significant.

Fig. S1

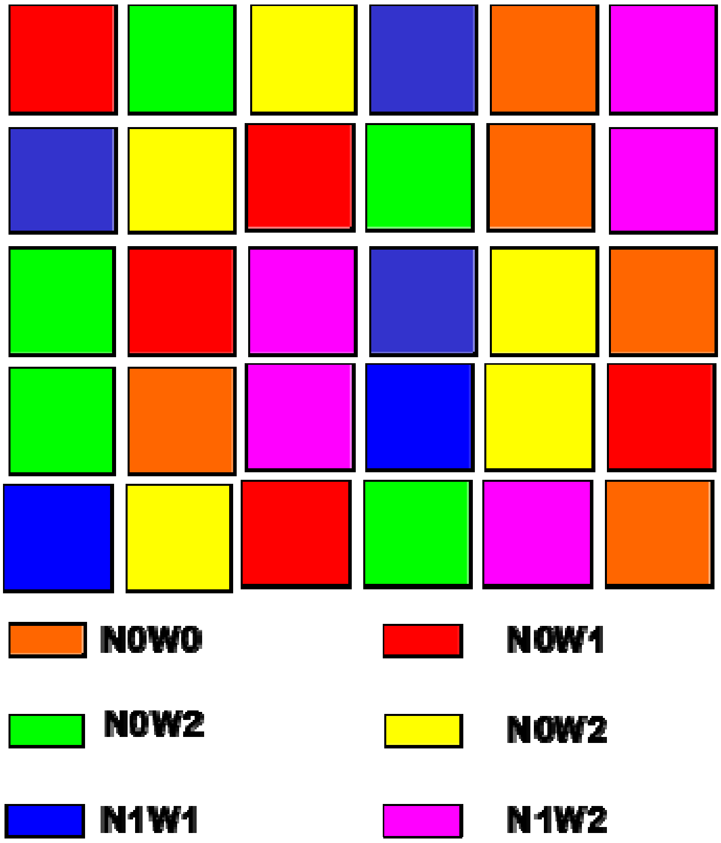

Fig. S2

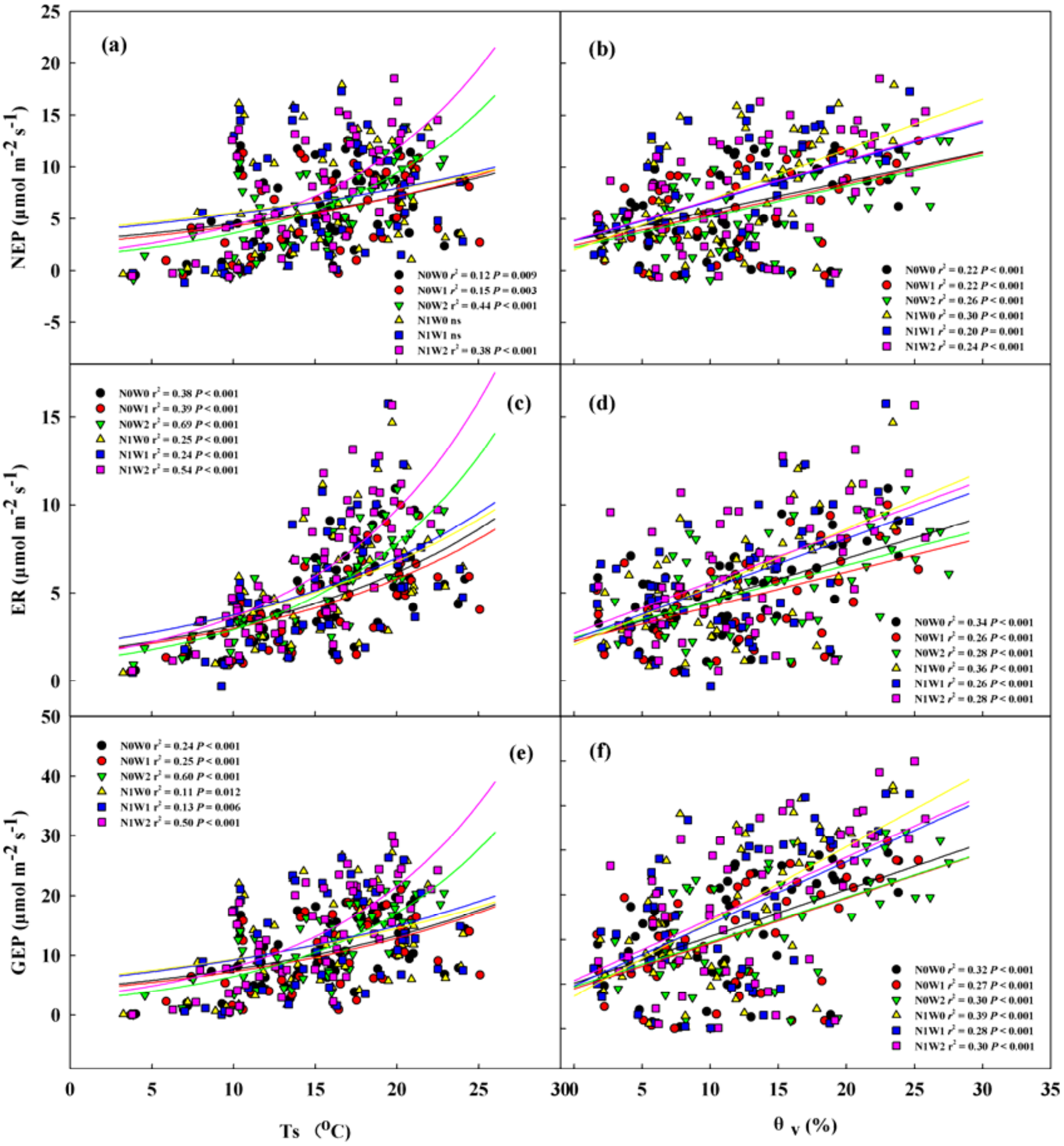

(a)
NEP (μmol m-2 s-1)
N0W0 r2 = 0.12 P = 0.009
N0W1 r2 = 0.15 P = 0.003
N0W2 r2 = 0.44 P < 0.001
N1W0 ns
N1W1 ns
N1W2 r2 = 0.38 P < 0.001
(b)
N0W0 r2 = 0.22 P < 0.001
N0W1 r2 = 0.22 P < 0.001
N0W2 r2 = 0.26 P < 0.001
N1W0 r2 = 0.30 P < 0.001
N1W1 r2 = 0.20 P = 0.001
N1W2 r2 = 0.24 P < 0.001
(c)
ER (μmol m-2 s-1)
N0W0 r2 = 0.38 P < 0.001
N0W1 r2 = 0.39 P < 0.001
N0W2 r2 = 0.69 P < 0.001
N1W0 r2 = 0.25 P < 0.001
N1W1 r2 = 0.24 P < 0.001
N1W2 r2 = 0.54 P < 0.001
(d)
N0W0 r2 = 0.34 P < 0.001
N0W1 r2 = 0.26 P < 0.001
N0W2 r2 = 0.28 P < 0.001
N1W0 r2 = 0.36 P < 0.001
N1W1 r2 = 0.26 P < 0.001
N1W2 r2 = 0.28 P < 0.001
(e)
GEP (μmol m-2 s-1)
N0W0 r2 = 0.24 P < 0.001
N0W1 r2 = 0.25 P < 0.001
N0W2 r2 = 0.60 P < 0.001
N1W0 r2 = 0.11 P = 0.012
N1W1 r2 = 0.13 P = 0.006
N1W2 r2 = 0.50 P < 0.001
(f)
N0W0 r2 = 0.32 P < 0.001
N0W1 r2 = 0.27 P < 0.001
N0W2 r2 = 0.30 P < 0.001
N1W0 r2 = 0.39 P < 0.001
N1W1 r2 = 0.28 P < 0.001
N1W2 r2 = 0.30 P < 0.001
Ts (0C)
θ v (%)

Table S1 Aboveground primary production (ANPP, g m^{-2}) and 0-50 cm belowground biomass (BGB, g m^{-2}) (mean ± 1SE) in control (N0W0), spring snow addition (N0W1), summer water addition (N0W2), nitrogen addition (N1W0), spring snow with nitrogen addition (N1W1) and summer water with nitrogen addition (N1W2) treatments in 2011, 2012 and 2013. Different letters indicate significant differences (*P* < 0.05) between means (n = 5).

	ANPP			BGB		
treatment	2011	2012	2013	2011	2012	2013
N0W0	332.6±33.0^{a}	478.8±71.0^{a}	293.9±8.6^{c}	927.1±111.4^{b}	974.4±90.2^{a}	2584.7±225.6^{ab}
N0W1	371.9±45.3^{a}	415.7±27.8^{ab}	306.8±27.7^{c}	982.0±82.9^{b}	1222.8±135.1^{a}	2344.3±103.6^{b}
N0W2	334.9±32.1^{a}	302.9±19.8^{b}	352.2±27.7^{c}	1306.2±140.2^{a}	1205.9±51.4^{a}	2914.6±63.6^{a}
N1W0	373.2±29.6^{a}	502.2±59.2^{a}	385.1±13.9^{bc}	943.0±40.6^{b}	987.2±73.4^{a}	2244.3±215.8^{b}
N1W1	403.7±39.9^{a}	511.1±30.6^{a}	457.7±29.7^{ab}	1174.8±118.9^{ab}	1231.1±146.3^{a}	2538.9±156.5^{ab}
N1W2	407.7±36.1^{a}	454.7±55.7^{a}	487.6±54.2^{a}	981.2±59.9^{b}	1115.0±100.5^{a}	2707.1±171.7^{ab}

Table S2 Values of soil ammonium and nitrate contents (mean ± 1SE, mg Kg^{-1}) in control (N0W0), spring snow addition (N0W1), summer water addition (N0W2), nitrogen addition (N1W0), spring snow with nitrogen addition (N1W1) and summer water with nitrogen addition (N1W2) treatments in 2011. Different letters indicate significant differences (*P* < 0.05) between means (n = 5).

Soil layer	treatment	NH_4-N			NO_3-N		
		2011.4	2011.7	2011.10	2011.4	2011.7	2011.10
0-10cm	N0W0	2.8±0.1[a]	3.3±1.2[a]	3.3±0.4[ab]	7.1±0.6[b]	5.8±0.7[c]	5.2±0.9[b]
	N0W1	2.9±0.6[a]	2.3±0.3[a]	2.2±0.5[b]	6.5±1.2[b]	7.2±1.4[c]	5.7±0.9[b]
	N0W2	3.4±0.5[a]	2.4±0.4[a]	2.5±0.5[b]	7.1±0.6[b]	11.8±3.6[bc]	3.6±0.7[b]
	N1W0	3.7±1.0[a]	4.9±2.0[a]	6.0±1.7[a]	11.2±1.3[a]	19.8±1.0[a]	12.5±3.1[a]
	N1W1	3.3±0.3[a]	2.1±0.4[a]	4.1±1.0[ab]	10.0±0.6[ab]	17.6±4.4[ab]	11.0±1.6[a]
	N1W2	3.9±1.0[a]	3.3±0.5[a]	3.8±0.7[ab]	8.9±2.2[ab]	18.0±1.3[ab]	6.2±0.3[b]
10-20cm	N0W0	3.7±0.3[b]	3.0±0.7[b]	4.4±0.8[a]	4.3±0.3[bc]	5.3±1.1[b]	1.0±0.1[b]
	N0W1	4.6±0.5[ab]	2.7±0.4[b]	2.9±0.4[a]	5.5±0.6[abc]	6.3±1.0[b]	1.4±0.3[b]
	N0W2	5.8±1.3[ab]	2.6±0.4[b]	2.8±0.5[a]	3.3±0.6[c]	6.2±0.9[b]	1.5±0.4[b]
	N1W0	6.8±1.1[a]	5.8±1.1[a]	3.9±0.8[a]	8.2±0.9[a]	12.0±0.7[a]	16.8±3.3[a]
	N1W1	4.1±0.6[ab]	2.8±0.7[b]	3.7±0.2[a]	7.5±1.0[ab]	13.4±3.4[a]	11.9±2.5[a]
	N1W2	6.2±1.0[ab]	3.8±0.6[ab]	3.6±0.3[a]	9.1±2.6[a]	13.7±1.6[a]	5.0±2.0[b]
20-30cm	N0W0	4.5±0.7[a]	2.1±0.5[a]	3.2±0.3[a]	2.9±0.4[c]	7.5±0.8[c]	0.7±0.1[d]
	N0W1	3.8±0.4[a]	2.7±0.5[a]	2.6±0.4[a]	3.9±0.9[bc]	7.9±1.2[bc]	0.6±0.2[d]
	N0W2	3.7±0.7[a]	2.3±0.4[a]	2.5±0.4[a]	2.6±0.7[c]	5.9±0.6[c]	1.2±0.28[cd]
	N1W0	4.3±0.5[a]	3.0±0.5[a]	3.3±0.4[a]	11.8±2.2[a]	12.4±1.3[a]	10.7±2.3[a]
	N1W1	3.7±0.4[a]	4.0±1.7[a]	3.2±0.3[a]	9.3±2.3[ab]	11.4±1.1[ab]	9.1±1.6[ab]
	N1W2	5.4±1.2[a]	3.6±0.3[a]	3.3±0.3[a]	9.6±3.9[ab]	11.4±2.2[ab]	5.1±1.9[bc]
